# Epigenetic regulation of lncRNA connects ubiquitin-proteasome system with infection-inflammation in preterm births and preterm premature rupture of membranes

**DOI:** 10.1186/s12884-015-0460-0

**Published:** 2015-02-15

**Authors:** Xiucui Luo, Jing Pan, Leilei Wang, Peirong Wang, Meijiao Zhang, Meilin Liu, Ziqing Dong, Qian Meng, Xuguang Tao, Xinliang Zhao, Julia Zhong, Weina Ju, Yang Gu, Edmund C Jenkins, W Ted Brown, Qingxi Shi, Nanbert Zhong

**Affiliations:** 1 Center of Translational Medicine for Maternal and Children’s Health, Lianyungang Maternal and Children’s Hospital, Lianyungang, Jiangsu China; 2 New York State Institute for Basic Research in Developmental Disabilities, Staten Island, NY USA; 3 Peking University Center of Medical Genetics, Beijing, China; 4 Children’s Hospital of Shanghai Affiliated to Shanghai Jiaotong University, Shanghai, China; 5 Hunter College High School, New York, USA; 6 Chinese Alliance of Translational Medicine for Maternal and Children’s Health, Beijing, China; 7 March of Dimes Global Network of Maternal and Infant Health, March of Dimes Foundation, White Plains, USA; 8 Department of Human Genetics, New York State Institute for Basic Research in Developmental Disabilities, 1050 Forest Hill Road, Staten Island, NY 10314 USA

**Keywords:** Preterm birth (PTB), Preterm premature rupture of membrane (PPROM), Long non-coding RNA (lncRNA), mRNA, Pathogenic mechanism

## Abstract

**Background:**

Preterm premature rupture of membranes (PPROM) is responsible for one third of all preterm births (PTBs). We have recently demonstrated that long noncoding RNAs (lncRNAs) are differentially expressed in human placentas derived from PPROM, PTB, premature rupture of the membranes (PROM), and full-term birth (FTB), and determined the major biological pathways involved in PPROM.

**Methods:**

Here, we further investigated the relationship of lncRNAs, which are differentially expressed in spontaneous PTB (sPTB) and PPROM placentas and are found to overlap a coding locus, with the differential expression of transcribed mRNAs at the same locus. Ten lncRNAs (five up-regulated and five down-regulated) and the lncRNA-associated 10 mRNAs (six up- and four down-regulated), which were identified by microarray in comparing PPROM vs. sPTB, were then validated by real-time quantitative PCR.

**Results:**

A total of 62 (38 up- and 24 down-regulated) and 1,923 (790 up- and 1,133 down-regulated) lncRNAs were identified from placentas of premature labor (sPTB + PPROM), as compared to those from full-term labor (FTB + PROM) and from premature rupture of membranes (PPROM + PROM), as compared to those from non-rupture of membranes (sPTB + FTB), respectively. We found that a correlation existed between differentially expressed lncRNAs and their associated mRNAs, which could be grouped into four categories based on the gene strand (sense or antisense) of lncRNA and its paired transcript. These findings suggest that lncRNA regulates mRNA transcription through differential mechanisms. Differential expression of the transcripts PPP2R5C, STAM, TACC2, EML4, PAM, PDE4B, STAM, PPP2R5C, PDE4B, and EGFR indicated a co-expression among these mRNAs, which are involved in the ubiquitine-proteasome system (UPS), in addition to signaling transduction and beta adrenergic signaling, suggesting that imbalanced regulation of UPS may present an additional mechanism underlying the premature rupture of membrane in PPROM.

**Conclusion:**

Differentially expressed lncRNAs that were identified from the human placentas of sPTB and PPROM may regulate their associated mRNAs through differential mechanisms and connect the ubiquitin-proteasome system with infection-inflammation pathways. Although the detailed mechanisms by which lncRNAs regulate their associated mRNAs in sPTB and PPROM are yet to be clarified, our findings open a new approach to explore the pathogenesis of sPTB and PPROM.

## Background

Preterm premature rupture of membranes (PPROM), which occurs in one-third of all preterm births (PTBs), is one of the major causes of prematurity [1]. It accounts for a disproportionate amount of perinatal morbidity and mortality [2,3]. PPROM has been associated with several pathologic processes [4,5]. In addition, preterm contractions can lead to separation of the amnion and choriodecidua, with an overall reduction in membrane tensile strength, while cervical dilation can result in exposure of the membranes to vaginal microorganisms and reduce underlying tissue support [6]. The molecular mechanisms, including epigenomic mechanisms, underlying sPTB and PPROM are not yet clearly understood.

Currently, high-throughput transcriptomic research has indicated that eukaryotic genomes transcribe up to 90% of the genomic DNA [7]. Except for 1–2% of these transcripts encoding for proteins, the vast majority are transcribed as noncoding RNAs (ncRNAs) [7,8]. Although ncRNAs are defined by the lack of a protein-coding sequence, they can play important roles in a variety of biological processes [9,10]. ncRNAs are grouped into two major categories according to their lengths: the short noncoding RNAs, which include microRNAs (miRNAs) as well as other non-coding transcripts of less than 200 nucleotides (nts), and the more recently described long noncoding RNAs (lncRNAs) that are longer than 200 nt [11,12]. miRNAs, the most widely studied class of short ncRNAs, mediate post-transcriptional gene silencing by controlling the translation of mRNA into protein [13,14] and are involved in regulating proliferation, differentiation, apoptosis, and development [15]. The disruption of expression of miRNAs has been found in many human diseases including cancers, neurological disorders, and cardiovascular disorders [15]. For example, miR-15 and miR-16 are deregulated in B cell chronic lymphocytic leukemia [16]; miR-206 deficiency accelerates amyotrophic lateral sclerosis [17]; and miR-1, which is involved in heart development, has been linked with arrhythmias through down-regulating expression of ion channel genes [18,19]. In addition, the disruption of other classes of short ncRNAs, such as small nucleolar RNAs (snoRNAs) and piwi-interacting RNAs (piRNAs), can also lead to different human diseases [15]. For example, the germline homozygous 2 bp (TT) deletion of the snoRNA U50 is associated with prostate cancer development [20], and overexpression of the piRNAs piwiL1 and piwiL2 is involved in somatic tumors [21-23]. Increasing evidence has revealed that lncRNAs can function as regulators of protein-coding gene expression and exert a variety of intrinsic functions in eukaryocytes [24]. In genomic contexts, lncRNAs can be transcribed from enhancers, promoters, introns of genes, pseudogenes, and antisense to genes [25]. They may influence almost every step in the life cycle of genes, and they carry out their biological roles through several different mechanisms, including regulating chromatin states and nuclear compartments [26-28], affecting the process of transcription [29-31], and mediating mRNA stability, splicing, and translation at the post-transcriptional level [32-34]. Recently, the disruption of lncRNAs was also found to be associated with different human diseases, as short ncRNAs were [35]. ANRIL is the antisense lncRNA of the *INK4* locus, and its altered activity could result in deregulated silencing of the *INK4* locus, which contributes to the initiation of several cancers [36-39]. The lncRNA MALAT-1 is associated with early-stage non–small cell lung cancer [40], which depends on its ability in regulating the alternative splicing through interaction with nuclear phosphoproteins [41,42]. In addition, the antisense lncRNA BACE1-AS, which is encoded by the opposite strand of the gene *BACE1*, can increase BACE1 mRNA stability and protein abundance at the post-transcriptional level, as has been shown to be true in Alzheimer’s disease [43]. Moreover, based on the recent expression analyses, multiple lines of evidence increasingly support the linkage of dysfunctions of lncRNAs to other human diseases, including neurodegenerative and psychiatric diseases [44], cardiovascular disease [45], and immune dysfunction and auto-immunity [30].

PPROM is a heterogeneous condition, although infection and inflammation are well documented as key etiological factors [38,39,46]. Earlier, we have hypothesized that the ncRNAs may play an epigenomic role in regulating the pathogenic development of PPROM. To test this, we applied microchip technology and identified more than a thousand placental lncRNAs. With these lncRNAs, we delineated more than 20 potential pathogenic pathways that are altered in PPROM [47]. Among these, we showed that the pathways of infection and inflammatory response, extra cellular matrix (ECM)-receptor interactions, apoptosis, actin cytoskeleton, and smooth muscle contraction are the major pathogenic pathways involved in the development of PPROM. Here, we further investigated whether the differentially expressed lncRNAs, which were identified from placentas derived from PPROM, regulate related mRNA transcription.

## Methods

### Ethics statement

The Ethics Committee of Lianyungang Maternal and Children’s Hospital reviewed and approved the research project. Written informed consent was obtained from the pregnant women who participated in this study. All material and data were previously coded and thus anonymous to the authors of this study.

### Placentas

A total of 40 placentas from age-matched pregnant women (25–30 years old) were divided into four groups of deliveries (10 placentas per group): sPTB, FTB, PPROM, and PROM, labeled as group A, B, C, and D, respectively. sPTB is defined as birth delivered at < 35 (= < 34^+6^) GW without premature rupture of amniochorionic membrane (PROM), FTB is defined as birth delivered at 39–40^+6^ GW without PROM; PPROM is defined as birth delivered at < 35 (= < 34^+6^) GW with PROM; and PROM is defined as birth delivered at 39–40^+6^ GW with premature rupture of the amniochorionic membrane before labor, which is the contraction of the uterus. Considering the epigenetic variation and high heterogeneity of PTB and PPROM, placentas were selected from pregnancies by using the criteria that there was no clinically recognized infection (no fever; no increase of white blood cell counts; no positive finding of amniotic fluid cultures; and no clinical intervention with antibiotics, steroid, or tocolytics during the pregnancy). Immediately following delivery, the placentas were flushed twice with 200 ml cold distilled water and dried with clean paper towels, sliced with a sterile scalpel into 1×1 cm^2^ cubes at a site 2 cm from the edge of the placenta, juxtaposed from the maternal side through the entire fetal membrane, quickly frozen in liquid nitrogen for 30 minutes, and stored at −80°C until use. The entire procedure was to be completed within 30 minutes after the placenta was delivered.

### Microarray hybridization

The Arraystar Human LncRNA Array v2.0 (www.arraystar.com) was employed in this study. This array covers 33,045 lncRNAs that were collected from the authoritative data sources RefSeq, UCSC Knowngenes, and Ensembl. RNA labeling and array hybridization were performed according to the Agilent One-Color Microarray-Based Gene Expression Analysis protocol (Agilent Technology, Santa Clara, CA) with minor modifications. Briefly, mRNA was purified from total RNA after removal of rRNA with mRNA-ONLY™ Eukaryotic mRNA Isolation Kit (Epicentre, Omaha, NE). Each sample was amplified and transcribed into fluorescent cRNA along the entire length of the transcripts without 3′ bias utilizing a random priming method. The labeled cRNAs were purified by RNeasy Mini Kit (Qiagen, Valencia, CA). The concentration and specific activity of the labeled cRNAs (pmol Cy3/μg cRNA) were measured by NanoDrop ND-1000. One μg of each labeled cRNA was fragmented by adding 5 μl 10 × blocking agent and 1 μl of 25 × fragmentation buffer, after which the mixture was heated at 60°C for 30 minutes, and 25 μl 2 × GE hybridization buffer was added to dilute the labeled cRNA. Fifty μl of hybridization solution was dispensed into the gasket slide and assembled to the lncRNA expression microarray slide. The slides were incubated for 17 hours at 65°C in an Agilent Hybridization Oven. The hybridized arrays were washed, fixed, and scanned by using the Agilent DNA Microarray Scanner (Agilent Technology, Santa Clara, CA). Agilent Feature Extraction software (version 11.0.1.1) was used to analyze the acquired array images. Quantile normalization and subsequent data processing were performed using the GeneSpring GX v12.1 software package (Agilent Technologies, Santa Clara, CA). After normalization of the raw data, lncRNAs and mRNAs that have flags (“All Targets Value”) were chosen for further data analysis. Differentially expressed lncRNAs and mRNAs with statistical significance between the two groups were identified through volcano plot filtering. Hierarchical clustering was performed using the Agilent GeneSpring GX software (Version 12.1). Both “GO analysis” and “Pathway analysis” were performed in the standard enrichment computation method. Results were also analyzed using the genetic and molecular interaction software GeneMANIA [48,49], an algorithm to indicate the co-relationship between these mRNA. The bio-functions and canonical pathways associated with our data were generated using the option of core-analysis in Ingenuity Pathway Analysis (IPA) (Ingenuity® Systems; http://www.ingenuity.com).

### Quantitative real-time PCR analysis

Total RNA was extracted from placentas, and cDNA was synthesized. The expression level of lncRNAs, as well as of lncRNA-associated mRNAs, was determined by quantitative real-time PCR (RT-qPCR). Primer sequences used in qPCR were listed in Tables 1 and 2. qPCR reactions were performed by the ABI7900 system (Applied Biosystems, CA) and SYBR green dye PCR master mix (SuperArray, CA). Glyceraldehyde 3-phosphate dehydrogenase (GAPDH) was used as an internal control, and lncRNAs’ or mRNAs’ values were normalized to GAPDH. Duplicated reactions were analyzed for each quantitative assay. For each lncRNA or mRNA, the result was finally reported as relative expression that was calculated relative to this control. All data were given in terms of relative expression of mean ± S.E. (N = 10). The data were subjected to one-way analysis of variance (one-way ANOVA) followed by an unpaired, two-tailed t-test. Differences were considered significant at *P* < 0.05 (labeled as “ * ” and extremely significant at *P* < 0.01 (labeled as “ ** ”).

**Table 1:** **Primers used for validation of lncRNA**

LncRNA	Primer
**GAPDH**	5′GAGTGGGTGTCGCTGTTGA3′
**ENST00000437593**	5′ GACCCATTCAAACTCTTTCACC3′
**BC017431**	5′ AAGCTGTCTGGTGCTGCTCTG3′
**BF328678**	5′ GGTAGAAGCGGATGAGTAGAAATAC3′
**ENST00000423797**	5′ GCAAGGAGAAGTGCCCAGAT3′
**BX483760**	5′ CCAGGCTGGTTTCAAACTCC3′
**AA451649**	5′ CTGAAGTGGAAGTTACAAGGAGGT3′
**DN918055**	5′ CTCCGCCATATTTGCCGTAC3′
**AX747492**	5′ CCACCGATGTCTGCCTATGTC 3′
**BG258490**	5′ CCAGTCTAGCCAACATAGCAAAC3′
**BF667001**	5′ GTTGTGGGTCGGTGTTTCC3′

**Table 2:** **Primers used for quantitating mRNA**

mRNA	Primers	Annealing (°C)	Amplicom (bp)
**GAPDH**	F:5′GGGAAACTGTGGCGTGAT3′	60	299
	R:5′GAGTGGGTGTCGCTGTTGA3′		
**ADAMTS15**	F:5′TGTGAAAGTCTGTGAGGAGGTGT3′	60	127
	R:5′CAGGAAGTCGGTGATGATGG3′		
**AQPEP**	F:5′GGACACGGAATACATGGTGC3′	60	137
	R:5′CCTGGTCGGTGTAGACGTTG3′		
**EML4**	F:5′TGAAGAGCCATGCAACGAGA3′	60	95
	R:5′AGCCAGGGTGTTAGGACGAG3′		
**NBPF10**	F:5′ATCAGCTTCGCCCTTTACG3′	60	82
	R:5′TGACTCCCATCTGGAACACC3′		
**PAM**	F:5′CCAGACCCGTAGTTCCTATTGA3′	60	96
	R:5′ACATGCAGAAGTATGTATCGGACT3′		
**PDE4B**	F:5′CAGAAAGCAAGACCAGGAAGC3′	60	185
	R:5′GGATAAGTTCCCGAAACTTAGTGC3′		
**PPP2R5C**	F:5′TTCATCGAGGTCTCGGGTAT3′	60	97
	R:5′CTTGAAGTGACAGCAGTGGG3′		
**STAM**	F:5′TGGCAGTGACACCAAAGATAGAG3′	60	141
	R:5′TAGCAAGGGATTAGACAGACGAA3′		
**TACC2**	F:5′GACACCTGTGATGAGTCCGTTG3′	60	149
	R:5′TTCTTGGCGGGCTTGTTTAG3′		
**TATDN1**	F:5′TGAAAACTGAAGCTAATTTGGAA3′	60	211
	R:5′ GCAGGGTTCATTTCTGTCTTT3′		

## Results

### Identification of differentially expressed lncRNAs in comparisons of group AC (PTB & PPROM) vs. group BD (FTB & PROM) and group CD (PPROM & PROM) vs. group AB (PTB & FTB)

**Figure 1:**
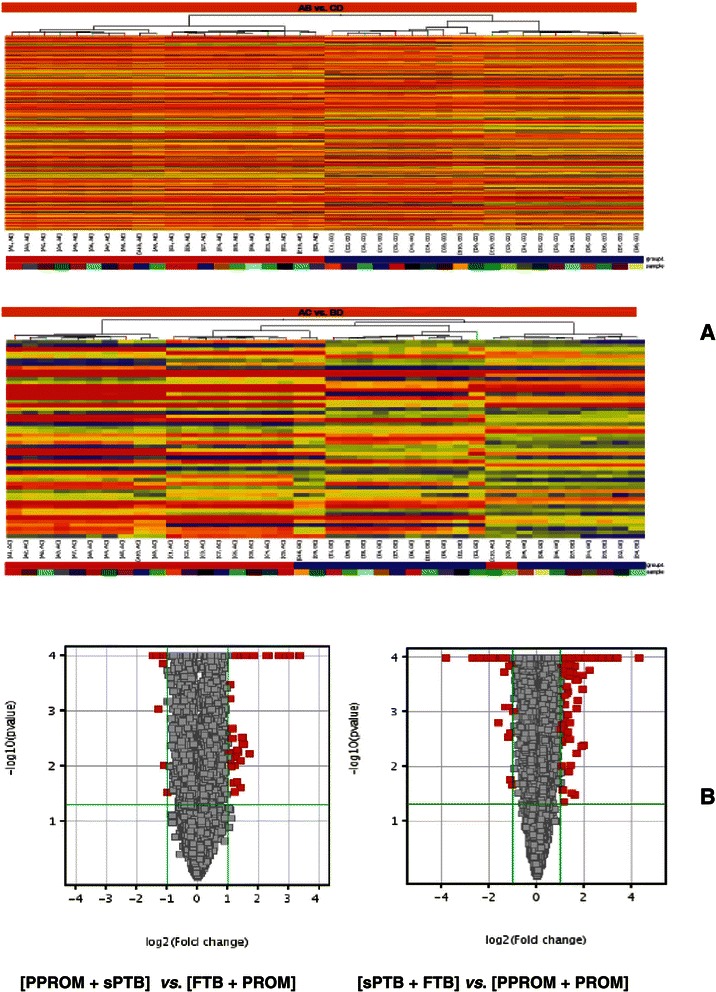
**Heat Maps and Volcano plots of two comparisons. (A)** Heat Maps: Differentially expressed lncRNAs for non-rupture of membrane vs. rupture of membrane (AB vs. CD) and preterm labor vs. full-term labor (AC vs. BD) were hierarchically clustered. “Red” indicates high relative expression, and “blue” indicates low relative expression. **(B)** Volcano Plots of two comparisons: X-axis is fold change (log 2) and Y-axis is *p* value (−log 10). Up-regulated (X axis > 0) or down-regulated (X axis < 0) lncRNAs (red squares) were identified in about the same number when fold change was set >2 folds [Log 2 (Fold change)] in PTB vs. PPROM.

Differential expression was both up- and down-regulated. A total of 62 (38 up- and 24 down-regulated) and 1,923 (790 up- and 1,133 down-regulated) lncRNAs were identified from group AC placentas compared to group BD, and from group CD compared to group AB, respectively (Figure 1A). To visualize differential expression between two different conditions, volcano plots (Figure 1B) were constructed by using fold-change (magnitude of change) values ≥ 2.0 and *p*-value < 0.05 as cut-offs, thus allowing visualization of the relationship between fold-change and statistical significance, which takes both magnitude of change and variability into consideration. The vertical lines correspond to 2.0-fold up and down, respectively, and the horizontal line represents a *p*-value of < 0.05. Therefore, the red point in the plot represents the differentially expressed lncRNAs with statistical significance. LncRNAs’ *p*-value < 0.0001, so those points were located in the axis Y = 4. Data of differentially expressed lncRNAs, generated by the microarray, has been deposited in Gene Expression Omnibus with an accession number GSE 50879 (http://www.ncbi.nlm.nih.gov/geo/info/linking.html).

### Metabolic pathways involved in group AC (PTB & PPROM) vs. group BD (FTB & PROM) and group CD (PPROM & PROM) vs. group AB (PTB & FTB)

**Figure 2:**
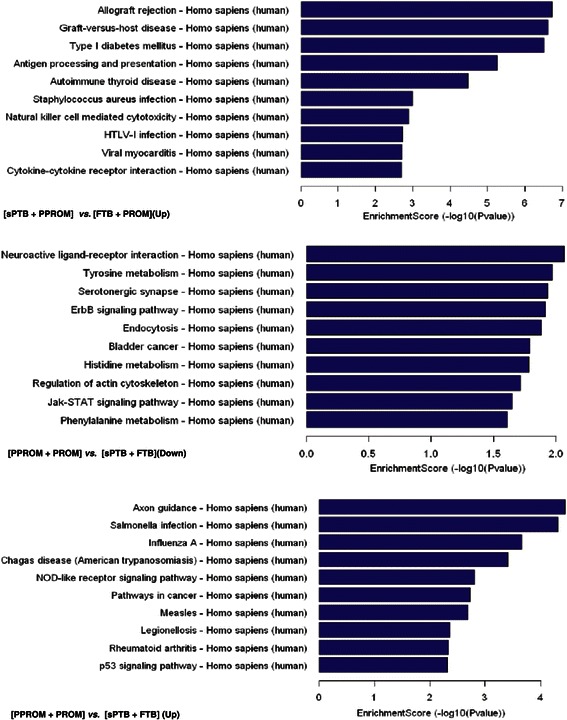
**Metabolic pathways identified from differentially expressed lncRNAs in PPROM.** Four groups of pathways (each group has up- and down-regulated) were characterized with KEGG functional analysis. Three p values, the EASE-score, Fisher-P value, and hypergeometric-P value, were integrated for the analysis. The bar plot shows the top enrichment score [−log10(Pvalue)] value of the significant enrichment pathway. If there were more than 10 pathways whose enrichment score is > 0.05, only the top 10 pathways are presented here. Three groups—AC *vs*. BD, up-regulation; CD *vs*. AB, up-regulation; and CD *vs*. AB, down-regulation—are shown here, with no down-regulation for AC *vs*. BD because few pathways could be identified by KEGG. The higher enrichment score indicates that more lncRNA molecules are involved in this pathway.

A functional analysis of mapping genes to KEGG (Kyoto Encyclopedia of Genes and Genomes, www.genome.jp/kegg/) pathways was performed with a *p*-value cut-off of 0.05. The *p*-value denotes the significance of the Pathway correlated to the conditions. The lower the *p*-value, the more significant is the Pathway. The up-regulated and down-regulated pathways with the Top 10 scores in group AC *vs*. group BD and group CD *vs*. group AB are shown in Figure 2, respectively.

### Quantitative real-time PCR validation of differentially expressed lncRNAs and their associated mRNAs

In this study, 10 samples from each group were analyzed, which gave a total of 40 samples. To address the tissue/cell heterogeneity, we randomly selected five loci and performed an intragroup analysis. Our results showed that there was no significant difference (p > 0.05) among the 10 samples, but they did show intergroup differences that were statistically significant.

**Figure 3:**
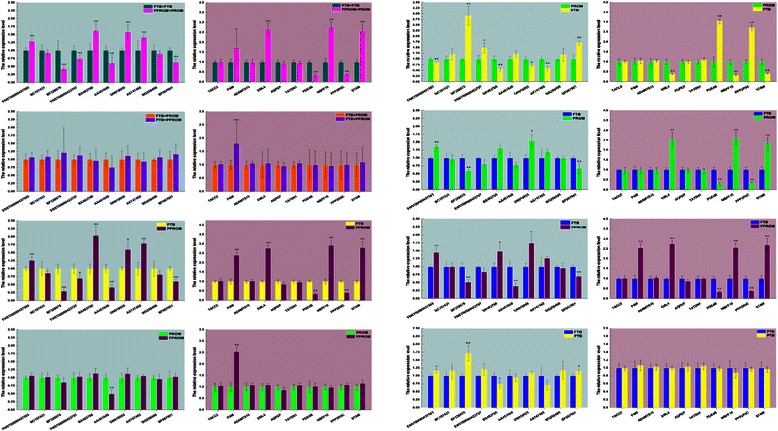
**Validation of lncRNAs.** RT-qPCR was applied to validate differentially expressed lncRNAs among eight pairs for comparison. Differential expression was studied for lncRNAs and compared to that of mRNAs. Labels of lncRNA names in the left panels correlate to those of the mRNA names in the right panels, e.g., ENST00000437593 of lncRNA correlates to mRNA TACC2, and BF667001 of lncRNA correlates to mRNA STAM. Significant levels were indicated by *(*p* < 0.05) and **(*p* < 0.01).

Ten paired lncRNAs and their associated mRNAs (PPP2R5C, STAM, TACC2, EML4, PAM, TATDN1, NBPF10, ADAMTS15, AQPEP, and PDE4B) with different expression patterns were selected from microarray data (Table 3). The selection was based on the following: 1) differentially expressed lncRNAs are natural antisense, 2) the sense strand of DNA has been transcribed into mRNA that is differentially expressed, and 3) the function of the gene is related to pathogenic pathway(s) that we have identified earlier from PTB and PPROM [47] or from this study as described above. RT-qPCR was used for validation of group PPROM compared to sPTB, PROM, and FTB; group sPTB compared to FTB and PROM; group PROM compared to FTB; the group of preterm labor [PPROM + sPTB] compared to full-term labor [PROM + FTB]; and the group of rupture of membrane [PPROM + PROM] compared to non-rupture of membrane [sPTB + FTB], respectively (Figure 3). The results of qPCR are summarized in Table 4. These comparisons revealed that, among 54 differentially expressed loci by microarray, one lncRNA (BC107431 in PPROM *vs*. sPTB) and seven mRNAs (ADAMTS15 in PPROM *vs*. sPTB; TATDN1 in PPROM *vs*. sPTB, PPROM *vs*. PROM, and [PPROM + PROM] *vs*. [sPTB + FTB]; AQPEP in PPROM *vs*. sPTB and sPTB *vs*. FTB; and TACC2 in PPROM *vs*. sPTB) by qPCR did not match the results of microarray, while 46 out of 54 (85.2%) were consistent with the results of microarray.

**Table 3:** **Correlation of differentially expressed lncRNAs with associated mRNAs detected by microarray**

		PPROM vs. sPTB		PPROM vs. PROM		PPROM vs. FTB		sPTB vs. FTB		PROM vs. FTB		sPTB vs. PROM		[PPROM + sPTB] vs. [FTB + PROM]		[PPROM + PROM] vs. [sPTB + FTB]	
lncRNA	mRNA	lncRNA	mRNA	lncRNA	mRNA	lncRNA	mRNA	lncRNA	mRNA	lncRNA	mRNA	lncRNA	mRNA	lncRNA	mRNA	lncRNA	mRNA
**BF328678**	**ADAMTS15**	**Down**	**Down**														
**BG258490**	**PPP2R5C**	**Down**	**Down**					**Down**	**Down**							**Down**	**Down**
**AA451649**	**TATDN1**	**Down**	**Up**	**Up**	**Down**											**Down**	**Up**
**BF667001**	**STAM**	**Down**	**Up**	**Up**	**Down**			**Down**	**Up**	**Down**	**Up**					**Down**	**Up**
**ENST00000423797**	**EML4**	**Down**	**Up**														
**AX747492**	**NBPF10**	**Up**	**Up**	**Down**	**Down**					**Up**	**Up**					**Up**	**Up**
**BC107431**	**PAM**	**Up**	**Up**														
**BX483760**	**AQPEP**	**Up**	**Up**			**Down**	**Up**	**Up**	**Up**								
**DN918055**	**PDE4B**	**Up**	**Down**	**Down**	**Up**			**Up**	**Down**	**Up**	**Down**					**Up**	**Down**
**ENST00000437593**	**TACC2**	**Up**	**Down**

**Table 4:** **Correlation of differential expression of lncRNAs with associated mRNAs detected by RT-qPCR**

Seqname		PPROM vs. sPTB		PPROM vs. PROM		PPROM vs. FTB		sPTB vs. FTB		PROM vs. FTB		sPTB vs. PROM		[PPROM + sPTB] vs. [FTB + PROM]		[PPROM + PROM] vs. [sPTB + FTB]	
lncRNA	mRNA	lncRNA	mRNA	lncRNA	mRNA	lncRNA	mRNA	lncRNA	mRNA	lncRNA	mRNA	lncRNA	mRNA	lncRNA	mRNA	lncRNA	mRNA
**BF328678**	**ADAMTS15**	**down**	**up**	**up**	**up**	**up**	**up**	**down**	**up**	**down**	**down**	**down**	**Up**	**up**	**up**	**down**	**down**
**BG258490**	**PPP2R5C**	**down**	**down**	**up**	**up**	**up**	**down**	**down**	**down**	**down**	**down**	**down**	**Up**	**up**	**up**	**down**	**down**
**AA451649**	**TATDN1**	**down**	**down**	**up**	**up**	**down**	**up**	**down**	**up**	**down**	**down**	**down**	**Up**	**down**	**up**	**down**	**down**
**BF667001**	**STAM**	**down**	**up**	**up**	**down**	**up**	**down**	**down**	**up**	**down**	**up**	**up**	**Up**	**up**	**up**	**down**	**up**
**ENST00000423797**	**EML4**	**down**	**up**	**up**	**down**	**up**	**down**	**down**	**up**	**down**	**up**	**up**	**Up**	**up**	**up**	**down**	**up**
**AX747492**	**NBPF10**	**up**	**Up**	**down**	**down**	**down**	**down**	**up**	**up**	**up**	**up**	**up**	**Down**	**down**	**down**	**up**	**up**
**BC107431**	**PAM**	**down**	**Up**	**up**	**up**	**up**	**up**	**down**	**up**	**down**	**up**	**up**	**Up**	**up**	**up**	**down**	**up**
**BX483760**	**AQPEP**	**up**	**down**	**down**	**up**	**down**	**up**	**up**	**down**	**up**	**up**	**up**	**Down**	**down**	**down**	**up**	**down**
**DN918055**	**PDE4B**	**up**	**down**	**down**	**up**	**up**	**down**	**up**	**down**	**up**	**down**	**up**	**Up**	**up**	**down**	**up**	**down**
**ENST00000437593**	**TACC2**	**up**	**Up**	**down**	**up**	**up**	**down**	**up**	**up**	**up**	**down**	**up**	**Up**	**up**	**up**	**up**	**up**

### The target mRNAs of IncRNA interaction relationship analysis by GeneMANIA

**Figure 4:**
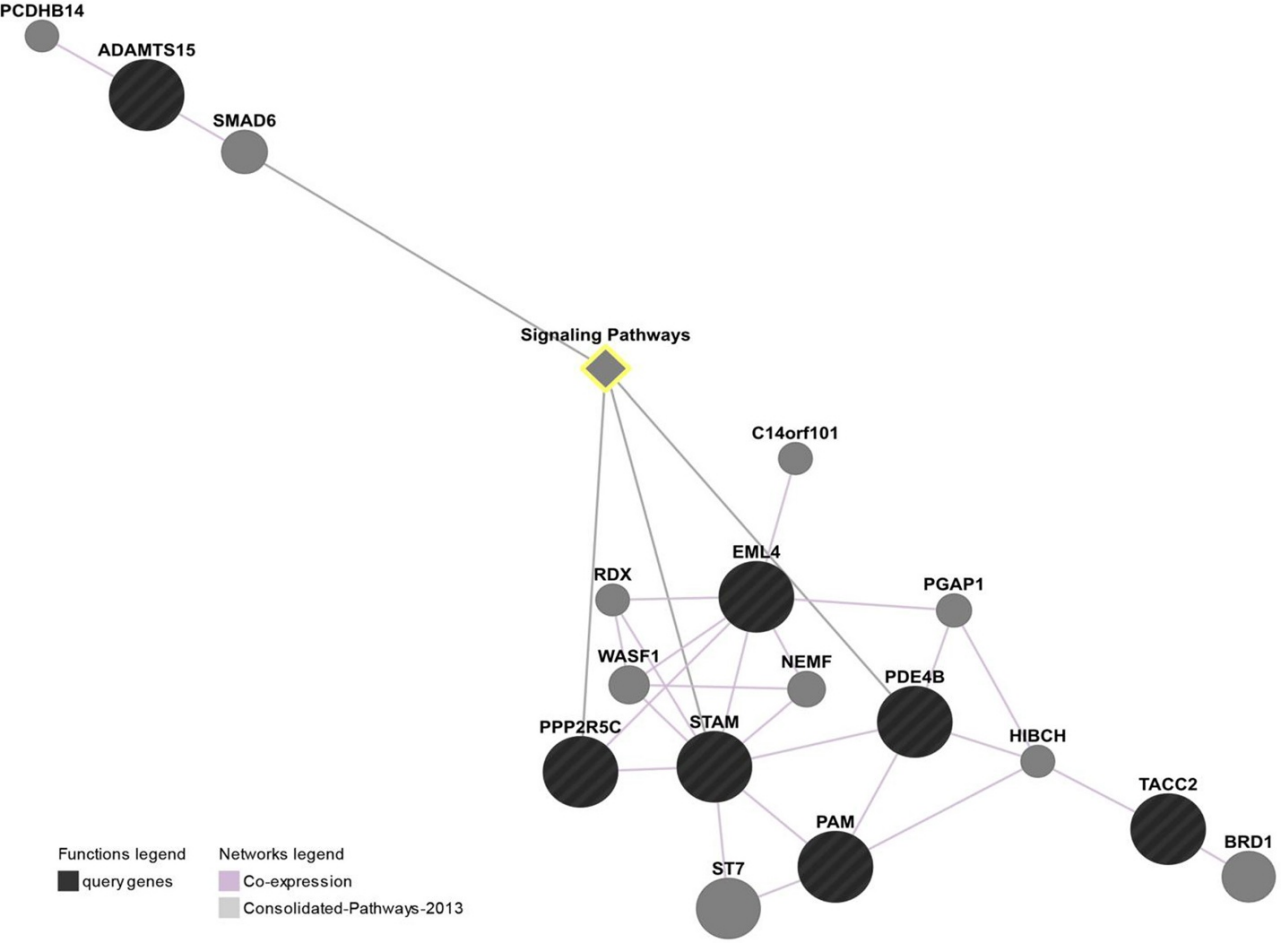
**Functional network of 10 mRNA obtained by GeneMANIA analysis.** Color representations: purple co-expression, dark referred to the target mRNAs. The core network of co-expression including PPP2R5C, STAM, TACC2, EML4, PAM, and PDE4B genes can be clearly seen.

**Figure 5:**
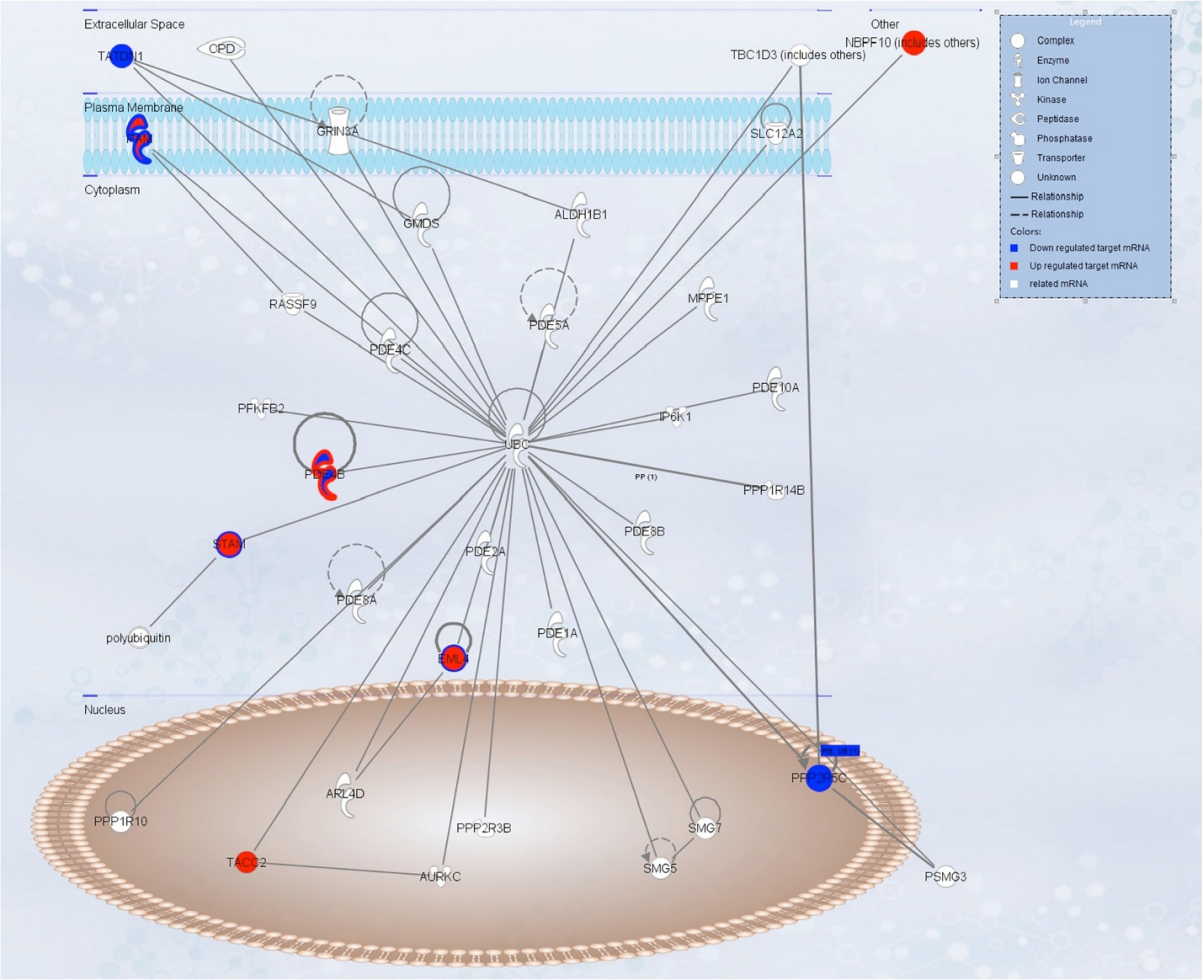
**The network graph of 8 mRNAs from 10 mRNAs obtained by IPA pathway analysis.** The red labels represent the target mRNAs referred to as functionally related molecules that may be co-expressed.

Ten differentially transcribed mRNAs regulated by IncRNA, including PPP2R5C, STAM, TACC2, EML4, PAM, TATDN1, NBPF10, ADAMTS15, AQPEP, and PDE4B, were subjected to GeneMANIA analysis. Six—PPP2R5C, STAM, TACC2, EML4, PAM, and PDE4B—were found to be in a functional network in terms of co-expression (Figures 4 and 5). The core pathway analysis of the 10 loci of lncRNA-mRNA pairs showed that the top canonical pathway of the mRNAs involved was cardiac β-adrenergic signaling. Eight targeted mRNAs (ADAMTS15, EML4, NBPF10, PAM, PDE4B, PPP2R5C, STAM, TACC2, and TATDN1) and 27 ubiquitin protease pathway–associated genes (www.ncbi.nlm.nih.gov/geo/info/linking.html, accession # GSE 50879) could be composed together to construct a network (Figure 4). These genes can be subcategorized into different families, including complex, enzyme, kinase, peptidase, ion channel, phosphatase, and transporter, and their top function and disease was DNA replication, recombination, and repair; nucleic acid metabolism and small molecule biochemistry. The cellular locations of these molecules can be mapped by IPA program, as shown in Figure 5, and imply a possible signal transduction from extracellular to nucleus.

## Discussion

This current study is a continuing investigation to explore the molecular mechanisms of lncRNA’s regulatory functions in both PTB and PPROM, based on our findings that lncRNAs are differentially expressed in the placentas of PTB and PPROM [47]. Histologically, the placenta contains both maternal (decidua) and fetal (chorionic villi and chorioamnionic membranes) tissues. Instead of using fetal membranes, which are essential for investigating the mechanisms directly related to the rupture of membrane, using the placenta may help us identify the lncRNAs that are differentially expressed in both maternal and fetal tissues and may provide a broad source of information related to the pathophysiological mechanisms underlying the premature contraction of the uterous, hemorrhage, and PROM, rather than only rupture of membrane. In addition, using the placenta as the study material may avoid the misidentifying of lncRNAs that maybe expressed in maternal tissues but not in fetal membranes. In our previous study, the lncRNAs from PPROM placentas and the pathogenic pathways they involved were identified based on microarray analysis [47]. However, the regulatory pattern between lncRNAs and their associated mRNAs in PPROM is still unknown. In the present study, the relationship between lncRNAs and their associated mRNAs was detected, which may help us further understand the molecular mechanism underlying PPROM.

PPROM, defined as rupture of the membranes before 37 weeks of gestation, contains two major pathogenic factors, membrane rupture and preterm labor [50,51]. In order to study these two factors individually, groups with preterm labor or membrane rupture were analyzed by microarray. Compared to the full-term group with or without premature rupture of membrane (group BD), preterm labor is now thought to be a syndrome initiated by multiple mechanisms, including infection or inflammation, uteroplacental ischaemia or haemorrhage, uterine over-distension, stress, and other immunologically mediated processes [52]. The up-regulated immune-related pathways in the preterm labor group (group AC) placentas further indicated that immunoreaction was one of the important risk factors in preterm labor. On the other hand, differentially expressed lncRNAs were identified from the premature membrane rupture group (group CD) placentas compared to the control group (group AB). Among the top ten up-regulated pathways, five were associated with microorganism infection, such as salmonella infection, influenza A, Chagas disease, measles, and legionellosis, and one (NOD-like receptor signaling pathway) was associated with the pathogen recognition process. One kind of etiologic factor linked to membrane rupture was microorganism infection and maternal or fetal host inflammatory response [5]. Infection-induced activation of membrane matrix-specific enzymes (matrix metalloproteinases, MMPs) has recently been shown to be associated with excessive collagen turnover and membrane weakening, leading to rupture [5]. The up-regulated pathways about microorganism infection and pathogen recognition further indicated that genital infections and inflammatory reactions were important pathological factors in membrane rupture. In addition, the up-regulated pathways in the membrane rupture group also contained the p53 signaling pathway, which was an important pathway leading to apoptosis and has been demonstrated to play an integral role in PPROM [4,53]. Up-regulated p53 signaling pathway might explain that microorganism infection may initiate membrane cell apoptosis through the p53 signaling pathway. The top ten down-regulated pathways in the premature membrane rupture group (group CD) were involved in amino acid, actin cytoskeleton regulation, and other signal transduction–related pathways. The down-regulation of these pathways might suggest that the placenta cells of the premature membrane rupture group have been in an apoptosis state and/or muscle contraction in a premature status, assuming the lncRNA(s) played a suppressing function for gene expression that resulted in increased transcription of mRNA(s).

It is possible for the lncRNAs to be located in the same strand or the antisense strand of their associated mRNA’s locus. Our microarray data showed that of the 10 paired lncRNAs and mRNAs studied, six pairs of locus TACC2, NBPF10, PPP2R5C, PAM, AQPEP, and PDE4B were located in the same strand (positive-strand), and the remaining four (ADAMTS15, EML4, STAM, and TATDN1) were in the antisense strands (Table 5). The differential expression pattern of lncRNAs between microarray data and RT-qPCR data among 27 comparisons (Table 3) showed 96% (26/27) agreement. However, the differential expression of the transcribed mRNA showed a lower rate (78%, 21/27) of agreement (Tables 3 and 4), suggesting that lncRNA is more stable than the coding mRNA. For example, both lncRNA and mRNA of ADAMTS15 were down-regulated in microarray assays; however, the lncRNA was down- but the mRNA was up-regulated in RT-qPCR. Nevertheless, Table 5 was constructed based on the qPCR results, in which we may see four categories of possible mechanisms by which lncRNA(s) regulate mRNA(s).

**Table 5:** **Regulation of lncRNAs on their associated mRNAs**

PPROM vs. PTB	Strand* (lncRNA/mRNA)	lncRNA	mRNA	Gene name	Possible regulation mechanism
**lncRNA and mRNA with same expression pattern and located in the same strand**	**+/+**	**↑**	**↑**	**TACC2**	**LncRNAs have miRNA-binding sites and compete for the miRNAs of mRNA.**
				**NBPF10**	
		**↓**	**↓**	**PPP2R5C**	
**lncRNA and mRNA with opposite expression patterns and located in the same strand**	**+/+**	**↓**	**↑**	**PAM**	**The product of gene is a bifunctional transcript, with one isoform as lncRNA and another as mRNA.**
		**↑**	**↓**	**AQPEP**	
				**PDE4B**	
**lncRNA and mRNA with opposite expression patterns and located in opposite strands**	**−/+**	**↓**	**↑**	**ADAMTS15**	**LncRNA is the host to miRNAs which regulate mRNA through RNAi.**
				**EML4**	
				**STAM**	
**ncRNA and mRNA with same expression pattern and located in opposite strands**	**+/−**	**↓**	**↓**	**TATDN1**	**LncRNA increases mRNA stability through masking miRNA site of mRNA.**

*: +, sense strand; −, antisense strand.

In the first category, lncRNAs and their associated mRNAs were located in the same strand, and both had the same expression pattern, which contains loci TACC2, NBPF10, and PPP2R5C. Recently, researchers have found that lncRNAs can compete for the miRNAs of mRNAs [25]. Because both lncRNA and mRNA are transcribed as the positive strand (or leading strand, labeled as “+” in Table 5), the possible mechanism of lncRNA regulating mRNA is that lncRNAs may function to bind to miRNAs, which protects mRNA from the miRNA targeting and repressing. Consequently, the transcript of mRNA is up-regulated, along with the up-regulation of lncRNA. TACC2, which belongs to a conserved family of centrosome- and microtubule-interacting proteins and encodes a protein that is concentrated at centrosomes throughout the cell cycle [54], represented an example of the phenomenon that when lncRNA is up-regulated, the mRNA at the same locus would be up-regulated, too. PPP2R5C represents another example in which both lncRNA and mRNA were down-regulated. PPP2R5C is one of the four major Ser/Thr phosphatases [55]. It was reported that a number of mammalian pseudogenes and lncRNAs have miRNA-binding sites in their 3′-UTRs and may therefore serve as “sponges” to sequester miRNAs away from miRNA-targets [56,57]. Considering that the lncRNAs and mRNAs of TACC2, NBPF10, and PPP2R5C were all in the sense strands and had the same expression pattern in regulating mRNA transcript in PPROM, the lncRNAs in the first category may regulate their associated mRNAs by competing for miRNA binding of mRNAs.

In the second category, lncRNAs and their associated mRNAs were also located in the same strand, but they had an opposite expression pattern, which contained loci of PAM, AQPEP, and PDE4B. In this group, lncRNA and mRNA might be transcribed bi-functionally. PDE4B is a member of the cyclic AMP (cAMP)-specific phosphodiesterase (PDE) family and encodes a protein that specifically hydrolyzes cAMP [58]. Considering that cAMP is one of the important second messengers regulating and mediating a number of cellular responses to extracellular signals, such as hormones, light, and neurotransmitters [59-61], the down-regulated PDE4B mRNA in the PPROM group might imply that the cAMP-related signal transduction was involved in the formation of PPROM. Another gene, AQPEP, encoding a novel membrane-bound aminopeptidase termed aminopeptidase Q, was expressed on extravillous trophoblast (EVT) during placentation [62,63]. It can degrade several placenta-derived peptides including kisspeptin-10 [62], which suppresses trophoblast as well as cancer migration [64-66] and could also be involved in the regulation of EVT migration. The down-regulation of AQPEP mRNA in the PPROM group might indicate that the pathogenesis of PPROM was associated with the placental lesion. In addition, PAM, encoding a multifunctional protein with catalytic activities to catalyze neuroendocrine peptides to active alpha-amidated products [67,68], was up-regulated. Further studies are needed to investigate the role of this gene in PPROM. A recent interesting discovery about lncRNA indicated that SRA is a bifunctional transcript, with one isoform functioning as an ncRNA and another as an mRNA that is translated into the protein SRAP [69,70], which may in turn antagonize the function of its noncoding counterpart [71]. Since lncRNAs and their associated mRNAs located in the same strand presented the opposite expression pattern, which is considered as bidirectional lncRNA in the second category, PAM, AQPEP, and PDE4B might possess similar transcripts as SRA in PPROM.

In the third category, lncRNAs and their associated mRNAs were located in different strands (lncRNAs in the antisense strand, and mRNAs in the sense strand) and had the opposite expression pattern, which contained genes of ADAMTS15, STAM, and EML4. ADAMTS15 encodes a member of the ADAMTS (a disintegrin and metalloproteinase with thrombospondin motifs) protein family. Proteins in the ADAMTS family share several distinct protein modules, including a propeptide region, a metalloproteinase domain, a disintegrin-like domain, and a thrombospondin type 1 motif [72,73]. Recently, researchers have found that degradation of extracellular matrix (ECM) by matrix metalloproteinases (MMPs) in fetal membrane plays significant roles in membrane rupture in PPROM [5,74]. Moreover, several other non-MMPs with a proteinase domain, such as serine proteases, cysteine proteases, and ADAMTS family members, can also break down amniochorion ECM substrates [74]. The up-regulated mRNA expression of ADAMTS15 in the PPROM group might indicate that ADAMTS15 probably functioned as MMPs participating in the regulation of collagenolysis and ECM degradation in PPROM [5]. Another interesting gene, STAM, encodes a member of the signal-transducing adaptor molecule family, which mediates downstream signaling of the cytokine-cytokine receptor interaction pathway [73]. Host inflammatory responses induced by different pro-inflammatory cytokines (including IL-1β, IL-6, and TNF-α) were closely associated with PPROM [5,75]. The up-regulated expression of STAM mRNA might imply that STAM was probably involved in PPROM through regulation of pro-inflammatory cytokine-cytokine receptor interaction. To gain further understanding of the regulation mechanism of STAM in PPROM, KEGG analysis of this gene was processed. The results revealed that STAM was involved in the JAK-STAT signaling pathway (data not shown but available upon request), which could be activated by IL-6 and cytokine receptor interaction. Moreover, depending on the connection with the MAPK or PI3K-Akt signaling pathways, STAM could participate in the regulation of apoptosis. Apoptosis has been demonstrated to play an integral role in PPROM, and IL-6 also has been proved to promote MMP activation or apoptosis of fetal membranes in PPROM. Therefore, STAM might regulate apoptosis of fetal membranes in PPROM by mediating downstream signaling of the IL-6 and cytokine receptor interaction pathway. The last gene in this category was EML4, which encodes a novel microtubule-associated protein belonging to the conserved family of EMAP-like proteins. EML4 has been demonstrated to be essential for microtubule formation [76]. Up-regulated expression of EML4 mRNA might imply that microtubule formation of the cytoskeleton in fetal membranes possibly was abnormal in PPROM. Because lncRNAs and mRNAs of ADAMTS15, STAM, and EML4 were located in opposite strands, lncRNAs in the third category could be complementary to their associated mRNAs. Although no evidence to date has demonstrated that lncRNAs can directly down-regulate their complementary mRNA through the RNAi-like pathway as miRNAs, the opposite expression pattern of antisense lncRNAs and sense mRNAs in this category could be explained by another new finding that lncRNAs could be the host genes for small RNAs [77]. For example, lncRNA H19 is host to miR-675 [78], and the imprinted Gtl2, anti-Rtl1, and Mirg RNAs are hosts to almost 50 miRNAs and 40 snoRNAs [79]. These lncRNAs could yield Dicer-dependent small RNAs and repress their complementary mRNAs through an RNAi-related pathway [77], which might be the mechanism whereby lncRNAs regulate mRNAs in this category.

In the fourth category, lncRNAs and their associated mRNAs were also located in opposite strands (lncRNAs in the antisense strand, and mRNAs in the sense strand), but they had the same expression pattern, which contained only one gene, TATDN1. TATDN1 encodes a protein known as a DNase domain-containing protein, which plays an important role in chromosomal segregation and cell cycle progression [80]. TATDN1 is a conserved nuclease in both prokaryotes and eukaryotes and has been found to play a role in apoptotic DNA fragmentation in yeast and *C. elegans* [81,82]. Although it is unclear whether TATDN1 in vertebrates also functions in apoptotic DNA fragmentation, the down-regulation of TATDN1 mRNA in the PPROM group at least suggested that TATDN1 might be associated with PPROM by regulating DNA fragmentation. A recent study about Alzheimer’s disease found that the antisense transcript of the Alzheimer-associated b-secretase-1 (BACE), known as BACEAS, increases BACE mRNA stability [43], most likely by masking the binding sites for miR-485-5p [83], which suggested that lncRNAs could interfere with miRNA-mediated mRNA destabilization. Because lncRNAs and mRNA of TATDN1 were located in the opposite strand, and both were down-regulated in the PPROM group, lncRNAs of TATDN1 might regulate its mRNA as BACEAS by masking the miRNA-binding sites of its mRNA in PPROM.

Although the 10 lncRNA-targeting mRNAs may be regulated by lncRNA with different mechanisms, six of them—PPP2R5C, STAM, TACC2, EML4, PAM, and PDE4B—were found to be directly co-expressed [84-87] in one network by GeneMANIA analysis, which indicated that their expression levels are similar across conditions. Three of these mRNAs, STAM, PPP2R5C, and PDE4B, and another co-expressed gene, SMAD6, were found to be involved in the signal transduction pathway (REACTOME REACT_111102.4) [88]. Signal transduction is a process in which extracellular signals elicit changes in cell state and activity. Depending on the cellular context, the signaling transduction pathway may impact cellular proliferation, differentiation, and survival. In these signaling pathways, PDE4B and PPP2R5C are involved in cardiac β-adrenergic signaling, which regulates the contraction of muscles. The earlier occurrence of uterine contraction was one of the main factors for PPROM. Therefore, the β-adrenergic signaling in uterine smooth muscles cells may be an important signaling pathway involved in PPROM. Further investigations should be pursued to explore how the mRNAs involved in this pathway are regulated by lncRNAs.

In addition to smooth muscle contraction, the ubiquitin C (UBC) network constructed by the eight lncRNA-targeted transcripts is shown in Figures 4 and 5. At the protein level, these targeted transcripts were found to interact with each other [89,90]. UBC is a polyubiquitin precursor. Conjugation of ubiquitin monomers or polymers can lead to various effects within a cell, depending on the residues to which ubiquitin is conjugated. Ubiquitination has been associated with protein degradation, DNA repair, cell cycle regulation, kinase modification, endocytosis, and regulation of other cell signaling pathways [91]. Protein degradation was one of the pathophysiological changes for PPROM. Although MMP was one of the clarified factors associated with PPROM, the mechanism of PPROM was still unclear. Protein degradation through the ubiquitin-proteasome system (UPS) is the major pathway of non-lysosomal proteolysis of intracellular proteins and played a modulation role in the immune and inflammatory responses. Our analysis provided a possible explanation for the multiple pathways involved in PPROM under the regulation of UPS. In our analysis, STAM has two ubiquitin-binding domains, Vps27/Hrs/Stam (VHS) and ubiquitin-interacting motif (UIM), at its N-terminus, and its function was as a signal-transducing adaptor, which mediates downstream signaling of the cytokine-cytokine receptor interaction pathway. PPP2R5C is involved in multiple pathways, and its dephosphorization may also play a central role in regulation of target proteins’ degradation *via* the UPS. Our finding that transcription of mRNA of STAM may be regulated by lncRNA opens a new approach to study the pathogenesis of sPTB and PPROM through the connection between UPS and infection-inflammation pathways.

## Conclusions

In conclusion, differentially expressed lncRNAs identified from the human placentas of PPROM might regulate their associated mRNAs through different mechanisms, as discussed above. The possible regulatory pattern between these lncRNAs and their associated mRNAs in PTB and PPROM might represent the potential molecular mechanism underlying PTB and PPROM. Although the detailed mechanisms through which lncRNAs regulate their associated mRNAs in sPTB and PPROM must be further clarified, our findings have identified a new way to explore the pathogenesis of sPTB and PPROM in the near future.
